# Combined Transcriptomic and Metabolomic Analysis Reveals Insights into Resistance of Arabidopsis *bam3* Mutant against the Phytopathogenic Fungus *Fusarium oxysporum*

**DOI:** 10.3390/plants11243457

**Published:** 2022-12-09

**Authors:** Eleni Kalogeropoulou, Konstantinos A. Aliferis, Sotirios E. Tjamos, Irene Vloutoglou, Epaminondas J. Paplomatas

**Affiliations:** 1 Laboratory of Mycology, Scientific Department of Phytopathology, Benaki Phytopathological Institute, 8 St. Delta Street, 145 61 Athens, Greece; 2 Laboratory of Pesticide Science, Agricultural University of Athens, 75 Iera Odos Street, 118 55 Athens, Greece; 3 Laboratory of Plant Pathology, Agricultural University of Athens, 75 Iera Odos Street, 118 55 Athens, Greece

**Keywords:** fusarium wilt, plant resistance, starch, maltose, β-amylases, trehalose, myo-inositol

## Abstract

The wilt-inducing strains of *Fusarium oxysporum* are responsible for severe damage to many economically important plant species. The most cost-effective and environmentally safe method for the management of Fusarium wilt is the use of resistant cultivars when they are available. In the present study, the Arabidopsis genotype with disruptions in the *β-amylase 3* (*BAM3*) gene, which encodes the major hydrolytic enzyme that degrades starch to maltose, had significantly lower susceptibility to *Fusarium oxysporum* f. sp. *raphani* (*For*) compared to wild-type (wt) plants. It showed the lowest disease severity and contained reduced quantities of fungal DNA in the plant vascular tissues when analyzed with real-time PCR. Through metabolomic analysis using gas chromatography (GC)–mass spectrometry (MS) and gene-expression analysis by reverse-transcription quantitative PCR (RT-qPCR), we observed that defense responses of Arabidopsis *bam3* mutants are associated with starch-degradation enzymes, the corresponding modification of the carbohydrate balance, and alterations in sugar (glucose, sucrose, trehalose, and myo-inositol) and auxin metabolism.

## 1. Introduction

Phytopathogenic strains of the *Fusarium oxysporum* species complex cause vascular wilt in a wide variety of plant species by directly penetrating roots and colonizing the vascular tissue, resulting in yield and economic losses [1]. Because of the pathogen’s lifestyle and the need for sustainable and environmentally friendly crop-management practices, the development of *F. oxysporum*-resistant cultivars is crucial [2]. Τechnological advances in genomics and bioinformatics enables the identification of genes controlling resistance within many pathogen–crop systems, providing a framework for improved selection efficiency [3].

Moreover, genetic studies have provided a new perspective, highlighting the relevance of primary metabolism in regulating plant defense against pathogens [4]. A number of investigations have shown that plants, including several important crops, reorganize starch metabolism to release energy, sugars, and derived metabolites contributing to plant fitness and survival upon stress [5]. In many cases, starch accumulates in response to stress, suggesting that the activation of starch degradation under stress is a common plant response and contributes to sugar accumulation [5].

In the model plant *Arabidopsis thaliana*, starch is degraded via an intricate network of reactions involving the synergistic action of multiple enzymes [6]. At first, starch is phosphorylated by two dikinases, the glucan water dikinase (GWD) and the phosphoglucan water dikinase (PWD), and the phosphoglucan phosphatase starch excess 4 [7]. Subsequently, the *β-amylase* (*BAM*) gene family (mainly *BAM3*) is a key regulator of starch hydrolysis into maltose in the chloroplast at night. Besides maltose, a range of short maltooligosaccharides emerge as byproducts that require further processing by chloroplastic glucanotransferase disproportionating enzyme (DPE1) to yield glucose and a spectrum of larger, linear oligosaccharides which can again be further metabolized by β-amylase [8,9]. In Arabidopsis, there are nine *BAM* genes: *BAM7* and *BAM8* encode proteins with N-terminal DNA-binding domains that are targeted to nuclei, where they function in regulating gene expression with no apparent catalytic activity as β-amylases; *BAM4* and *BAM9* encode catalytically inactive proteins located in plastids, but *BAM4* may play a role in regulating starch metabolism [7]. Of the five remaining *BAM* genes, *BAM3* alone is sufficient to degrade leaf starch completely, whereas *BAM1* can only partially degrade leaf starch. In contrast, *BAM2, BAM5*, and *BAM6* have no detectable effects on starch degradation or plant growth [7]. For complete starch degradation, BAM3 works synergistically with debranching enzymes, mainly the isoamylase 3 (ISA3), which are responsible for the hydrolysis of the a-1,6-branches of starch into short, soluble maltooligosaccharides [6]. A plastid-localized α-amylase (AMY3) may also play a role in starch degradation; it works synergistically with BAM1 in guard cells to efficiently degrade starch [5].

Numerous studies have shown that sugars play a key role in plant defense responses to various abiotic and biotic stress factors; they constitute the primary substrate providing energy and structural material for defense responses, and the carbon skeleton for the synthesis of defense compounds, including secondary metabolites such as flavonoids, stilbenes, and lignin [10]. In addition, carbohydrates, such as sucrose, glucose, fructose, and trehalose, represent metabolic-signaling molecules in host plant cells, which induce the expression of many defense-related genes [11]. Formela-Luboińska et al. [12] emphasized the role of sucrose and various monosaccharides as signaling compounds for the regulation of phytohormones, abscisic acid (ABA), ethylene, salicylic acid (SA), and its glucoside (SAG), as well as phenylalanine ammonia lyase (PAL), benzoic acid 2-hydroxylase (BA2H), and superoxide dismutase (SOD) activities in embryo axes of yellow lupine upon inoculation with the hemibiotrophic fungus *F. oxysporum* f. sp. *lupini*. Positive correlations were observed between the sugar levels and the postinfectious production of SA, SAG, ABA, and ethylene, as well as the increased activity of PAL, BA2H, and SOD, with all of these parameters participating in the defense responses of yellow lupine to *F. oxysporum*.

Despite evidence of the biological benefits of trehalose, relatively little is known about how trehalose and related metabolites function during plant–microbe interactions. The biosynthesis of trehalose in plants involves the generation of trehalose-6-phosphate (T6P) from glucose-6-phosphate and UDP-glucose by trehalose-6-phosphate synthase (TPS), and the subsequent dephosphorylation of T6P to trehalose by trehalose-6-phosphate phosphatase (TPP) [13]. The *A. thaliana* genome contains 11 *TPS* (*AtTPS1–11*) genes and 10 *TPP* (*AtTPPA–J*) genes [14]. Out of the 10 *TPPs* in Arabidopsis, *AtTPPA* and *AtTPPB* genes have been shown to encode active TPP enzymes [14], while the activity of the other TPPs remains to be determined.

Major plant hormones that regulate defense responses include salicylic acid (SA), jasmonic acid (JA), and ethylene (ET). Nevertheless, plant hormones, such as auxins, abscisic acid (ABA), cytokinins, gibberellins, and brassinosteroids, that have been thoroughly described to regulate plant development and growth, have recently emerged as key regulators of plant immunity [15]. Auxin is well-known for being involved in many aspects of plant growth and development, but recent findings have revealed that elevated levels of indole-3-acetic acid (IAA), the most common naturally occurring plant hormone of the auxin class, or enhanced auxin signaling, can also promote disease development in some plant–pathogen interactions [16]. Covalent conjugation of IAA with sugars or cyclic polyalcohols (myo-inositol), amino acids, or proteins, maintains a proper auxin homeostasis, while IAA-amino-acid linkages, such as IAA-aspartate (IAA-Asp), have been reported to be a source of free auxin or act as a signaling molecule during plant infection, as well as to modulate plant responses to abiotic stress effectors [17]. GH3 family members are reported to function in plant-defense signaling through the maintenance of auxin homeostasis; GH3 family proteins control auxin conjugation, resulting the suppression of auxin signaling, thus leading to a delicate balance between development and defense processes [18]. Arabidopsis AtGH3.2, AtGH3.3, AtGH3.4, AtGH3.6, AtGH3.9, and AtGH3.17, as well as grape VvGH3.1, have adenylation activity that enables conjugating amino acids for IAA, and AtGH3.5 shows adenylation activity for both IAA and SA [18]. Additionally, AtGH3.5 synthesizes camalexin, which acts as the major phytoalexin in *A.* thaliana [19]. Despite the fact that the GH3 proteins were found in many plant species, little is known about their function and the impact of their conjugates on plant defense against fungi.

Although the importance of starch degradation during abiotic stress in several plant species has been recorded, the overall available knowledge is still fragmentary, and there is little evidence for its role upon exposure to pathogens. Therefore, in the present study, we investigated the role of *bam3* in *A. thaliana* defense against *F. oxysporum* f. sp. *raphani* (*For*), as well as the contribution of sugar and auxin metabolism in the intricate network of induced defense responses of *bam3* plants against the Fusarium-wilt pathogen using combined transcriptomic and metabolomic analysis.

## 2. Results

### 2.1. Disruption of BAM3 Gene Increased Resistance to F. oxysporum f. sp. raphani

**Figure 1:**
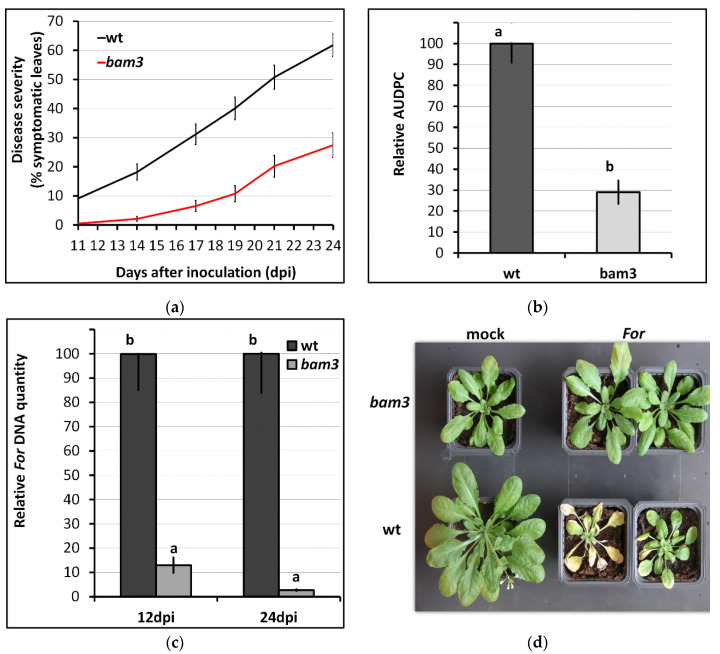
Disease severity in *Arabidopsis thaliana bam3* mutants and wt plants inoculated with *Fusarium oxysporum* f. sp. *raphani* (*For*), (**a**) periodically recorded for 24 days post inoculation (dpi) and expressed as the percentage of wilting leaves out of the total number of leaves present on each plant in 3 independent bioassays (total of 90 plants); (**b**) expressed as a percentage of the maximum possible area under the disease-progress curve for the whole period of the experiment, referred to as relative AUDPC; (**c**) relative quantification of *For* DNA in wt and *bam3* plants. The fungal DNA levels were estimated by qPCR of the total DNA, isolated from the aerial parts of 10 plants constituting one biological replicate per genotype and treatment, sampled at 12 and 24 dpi. The columns represent the means of three independent bioassays, with each bioassay consisting of 30 plants per genotype and treatment. The vertical bars indicate standard errors, and columns with different letters are statistically different according to Fisher’s LSD multiple-range test at *p* ≤ 0.05; (**d**) Fusarium-wilt symptoms caused on *For*-inoculated or mock *A. thaliana bam3* and wt plants at 21 dpi.

Visual assessment of Fusarium-wilt symptoms was performed from 11 days post inoculation (dpi) (first day of their appearance) to 24 dpi on root-infection assays of *A. thaliana* wt and *bam3* plants. The disease severity (% symptomatic leaves) was 62% in wt plants, while it was reduced to 27% in *bam3* mutants at 24 dpi (Figure 1a). Moreover, *bam3* plants showed less prominent symptoms (AUDPC 29%), while the disease severity progressed rapidly in wt plants (Figure 1b,d), implying that *bam3* mutants showed increased resistance to *For.* Estimation of the level of vascular fungal colonization by real-time PCR showed that *bam3* mutants showed 9% and 3% relative quantity of the pathogen at 12 and 24 dpi, respectively, compared to wt (Figure 1c).

### 2.2. Expression Analysis of Starch-Degradation-Related Genes in bam3 Mutant in Response to F. oxysporum f. sp. raphani Inoculation

To investigate the transcriptional regulation of β-amylolysis upon inoculation with *For* in resistant *bam3* mutants compared to wt plants, we performed a gene-expression analysis of the *BAM1*, *BAM2*, *BAM3,* and *BAM4* genes encoding the chloroplastic β-amylases. According to the results of preliminary microarray experiments (data not shown), the following genes encoding hydrolytic enzymes of starch degradation were also chosen for gene-expression analysis: *AMY3* encoding α-amylase 3, *ISA3* encoding the debranching enzyme isoamylase 3, and *DPE1* encoding the disproportionating enzyme 1.

**Figure 2:**
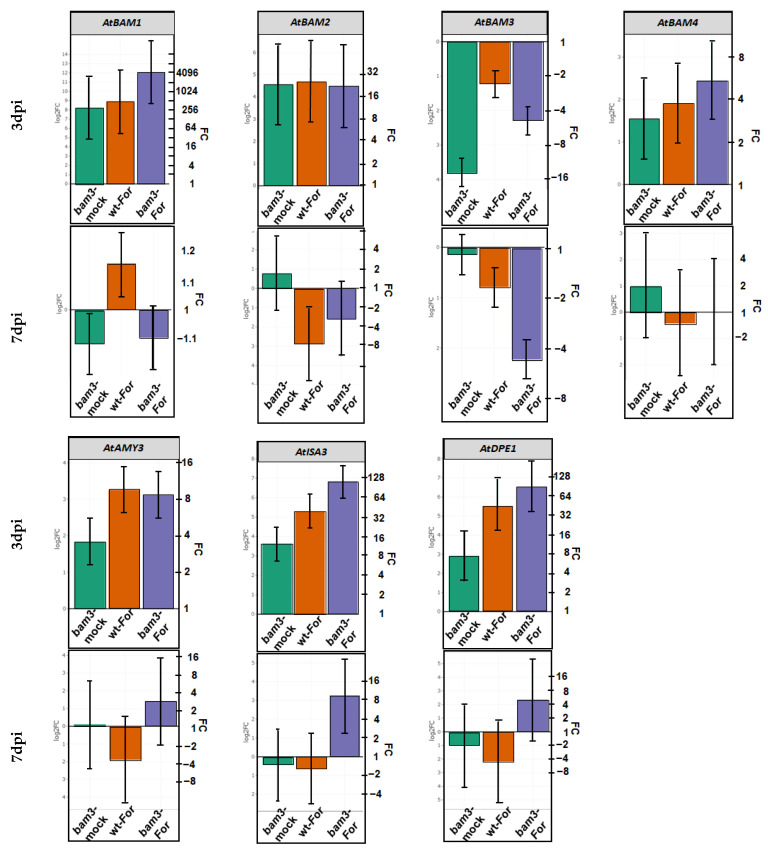
Comparative transcriptional analysis of *BAM1*, *BAM2*, *BAM3*, *BAM4*, *AMY3*, *ISA3*, and *DPE1* related to starch degradation. Fold change represents the relative difference in expression between each treatment (i.e., mock *bam3* plants, *For*-inoculated wt, and *For*-inoculated *bam3* plants) and control (i.e., wt mock plants) (x axis) at three and seven dpi. Three independent biological replicates with two technical replicates were analyzed, and *β-ACTIN2*, *β-ACTIN7*, and *β-ACTIN8* were used as reference genes. Bars indicate the 95% confidence interval of the estimates (2*SE) in all panels. Statistical analysis was performed with linear mixed models. Estimates for which the confidence interval includes the baseline value 0 (1 in fold-change scale) are not significantly different from the control at α = 5%.

The expression of *BAM3* was lower in mock mutants than in the corresponding wt plants, a reduction that was significant at three dpi (14-fold) but not at seven dpi (1.1-fold) (Figure 2). Inoculation with *For* reduced the relative levels of mRNAs of the gene in *bam3* plants 4.8-fold at both time points, while the reduction was lower in wt plants (2.3-fold and 1.7-fold at three and seven dpi, respectively) (Figure 2).

Contrariwise, *BAM1* was overexpressed 294-, 484-, and 4329-fold in mock *bam3* plants, *For*-inoculated wt, and *For*-inoculated *bam3* plants, respectively, compared to wt mock plants at three dpi, but was not altered significantly at seven dpi (Figure 2).

*BAM2* and *BAM4* were upregulated in mock *bam3* plants, *For*-inoculated wt, and *For*-inoculated *bam3* plants compared to wt mock plants without significant differentiation between the biological groups at three dpi. At seven dpi, *BAM4* was not significantly differentiated, while *BAM2* was downregulated 7.4- and 3-fold in inoculated wt and *bam3* plants, respectively (Figure 2).

Mock *bam3* plants showed induced expressions of *AMY3*, *ISA3,* and *DPE1* compared to wt mock plants at three dpi (3.6-, 12.4-, and 7.5-fold, respectively) (Figure 2). The above genes were also induced 2.4-, 9.2-, and 12.2-fold, respectively, in inoculated mutant plants compared to the noninoculated ones. At seven dpi, no differential gene expression was observed with the exception of *ISA3*, which was induced 9.6-fold in *For*-inoculated *bam3* plants compared to the mock mutant plants (Figure 2).

### 2.3. Maltose and Starch-Derived Glucose Contents in bam3 Mutant upon Inoculation with F. oxysporum f. sp. raphani

**Figure 3:**
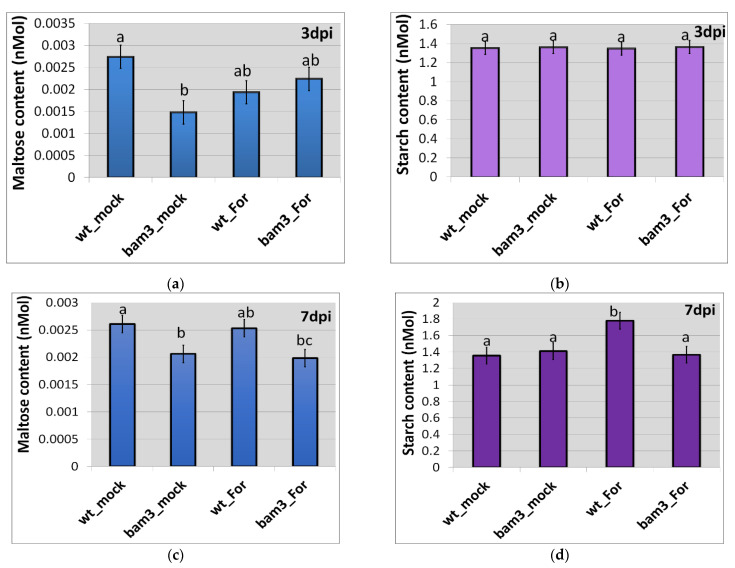
Quantification of maltose (**a**,**c**) and starch (**b**,**d**) content in the tissues of mock and inoculated with *Fusarium oxysporum* f. sp. *raphani* (*For*) *Arabidopsis thaliana* wt and *bam3* plants at three and seven dpi. Data are presented as means ± standard error (SE) of three independent biological replicates, with two technical replicates each. Different letters on columns indicate significant differences according to ANOVA and Fisher’s LSD multiple-range test at *p* ≤ 0.05.

To estimate the content of maltose and starch (starch-derived glucose) in the above-ground tissues of Arabidopsis *bam3* and wt plants, in the presence and absence of *For*, standard curves were initially constructed using known concentrations (0–50 nM) of standard solutions of maltose and starch, respectively. In the absence of the pathogen, *bam3* plants contained less maltose but the same amount of starch compared to wt plants at three and seven dpi (Figure 3a,b). The observed fluctuations in the maltose concentration in wt and *bam3* plants at three and seven days after *For* inoculation were not significant (Figure 3a). In addition, starch content was not altered among the biological groups, with the exception of *For*-inoculated wt plants, which showed the highest starch content at seven dpi (Figure 3b).

### 2.4. Metabolite Profiling of Inoculated and Noninoculated wt and bam3 Plants

**Figure 4:**
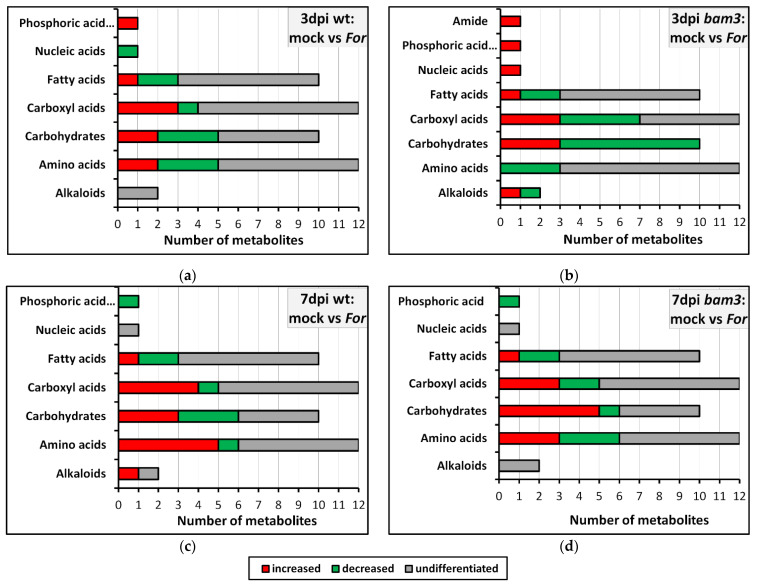
Classification of *Arabidopsis thaliana* wt and *bam3* metabolites into chemical groups in response to *Fusarium oxysporum* f. sp. *raphani (For)* inoculation. The total number of signatory metabolites, classified into increased, decreased, and undifferentiated in wt (**a**,**c**) and *bam3* (**b**,**d**) *For*-inoculated plants compared to mock plants at three (**a**,**b**) and seven (**c**,**d**) days post inoculation (dpi), are presented in the above graphs. For the chemical classification of metabolites, information was retrieved from the PubChem database.

Fluctuations in wt and *bam3* metabolomes in the absence and presence of *For* were recorded by GC/MS using bioinformatics software and species-specific metabolite databases. A total of 48 metabolites concerning the Arabidopsis metabolome were identified in response to *For* inoculation in wt and *bam3* plants at three and seven dpi, as well as in comparison of wt and *bam3* plants, mock or *For*-inoculated, at both time points. Differentially accumulated metabolites were identified for each comparison group by univariate and multivariate statistical analysis, with threshold values of VIP ≥ 1 and fold change ≥ 2 and ≤−2. Most metabolites showing differential accumulation belong to sugars, carboxyl acids, amino acids, and fatty acids (Figure 4). Specifically, three days after *For* inoculation, *bam3* plants showed changes in 10 carbohydrates (seven downregulated and three upregulated), seven carboxyl acids (four downregulated and three upregulated), three amino acids (downregulated), and three fatty acids (two downregulated and one upregulated), while the corresponding differentiations were less in wt plants (five carbohydrates, four carboxyl acids, five amino acids, and three fatty acids) (Figure 4a,b). It is worth mentioning that a significant change in the concentration was found in a small number of metabolites of *bam3* plants of other chemical groups related to plant metabolism, such as alkaloids (Figure 4b). At seven dpi, almost the same number of differentially produced metabolites was observed in wt and *bam3* plants, but they had significant variations in the amount of their production (Figure 4c,d).

### 2.5. KEGG Classification and Enrichment Analysis of Differential Metabolites

**Figure 5:**
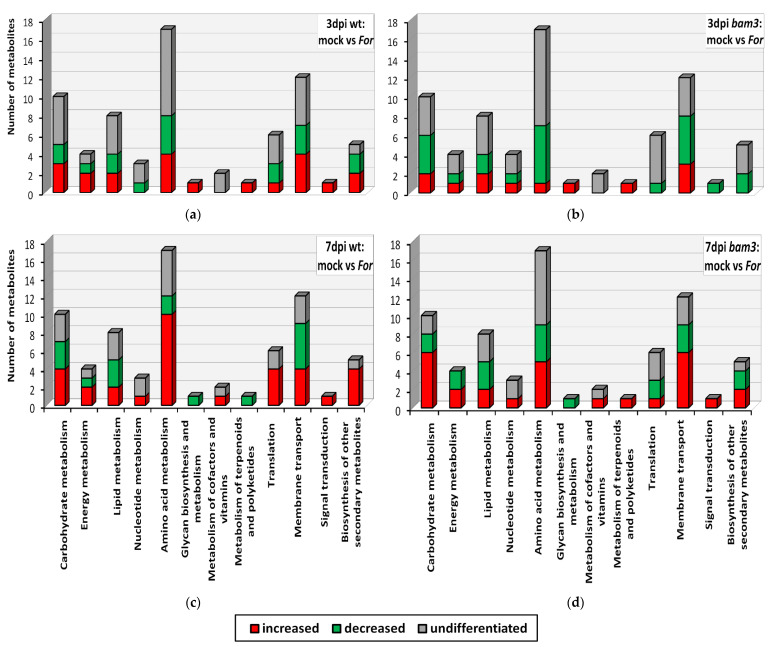
Classification of *Arabidopsis thaliana* wt and *bam3* signatory metabolites based on their participation in metabolic pathways, according to the KEGG database, after inoculation with *Fusarium oxysporum* f. sp. *raphani* (*For*). The number of increased, decreased, and undifferentiated metabolites in the comparison between mock and *For*-inoculated wt plants at three and seven dpi are presented in graphs (**a**) and (**c**), respectively. The number of increased, decreased, and undifferentiated metabolites in the comparison between mock and *For*-inoculated *bam3* plants at three and seven dpi are presented in graphs (**b**) and (**d**), respectively.

The 48 metabolites showing differential production in all comparison groups were mapped to the KEGG database to determine which metabolic pathways they might be associated with. Most of the metabolites were mapped to the metabolism of carbohydrates, membrane transport, amino acids, and lipids (Figure 5).

*For* inoculation caused decreased production in 36% of the differentially produced carbohydrates in *bam3* plant tissues at three dpi, (Figure 5b), while at seven dpi, 64% of them were increased (Figure 5d). Moreover, 25% and 41% of the differentially produced metabolites related to the membrane transport were increased and decreased in mutants, respectively, at three dpi, while 50% of them showed increased production; only 25% of them were decreased at seven dpi (Figure 5b,d). The amino acid metabolism of *bam3* and wt plants was also affected by fungal inoculation, since the production of seven and nine amino acids were differentiated at three and seven dpi, respectively, in mutant plants (Figure 5b,d), and eight and twelve amino acids were differentiated, at three and seven dpi, respectively, in wt plants (Figure 5a,c).

### 2.6. Metabolic Biomarkers in Response to F. oxysporum f. sp. raphani in bam3 Mutants

#### 2.6.1. Carbohydrates and Related Biomarkers

**Figure 6:**
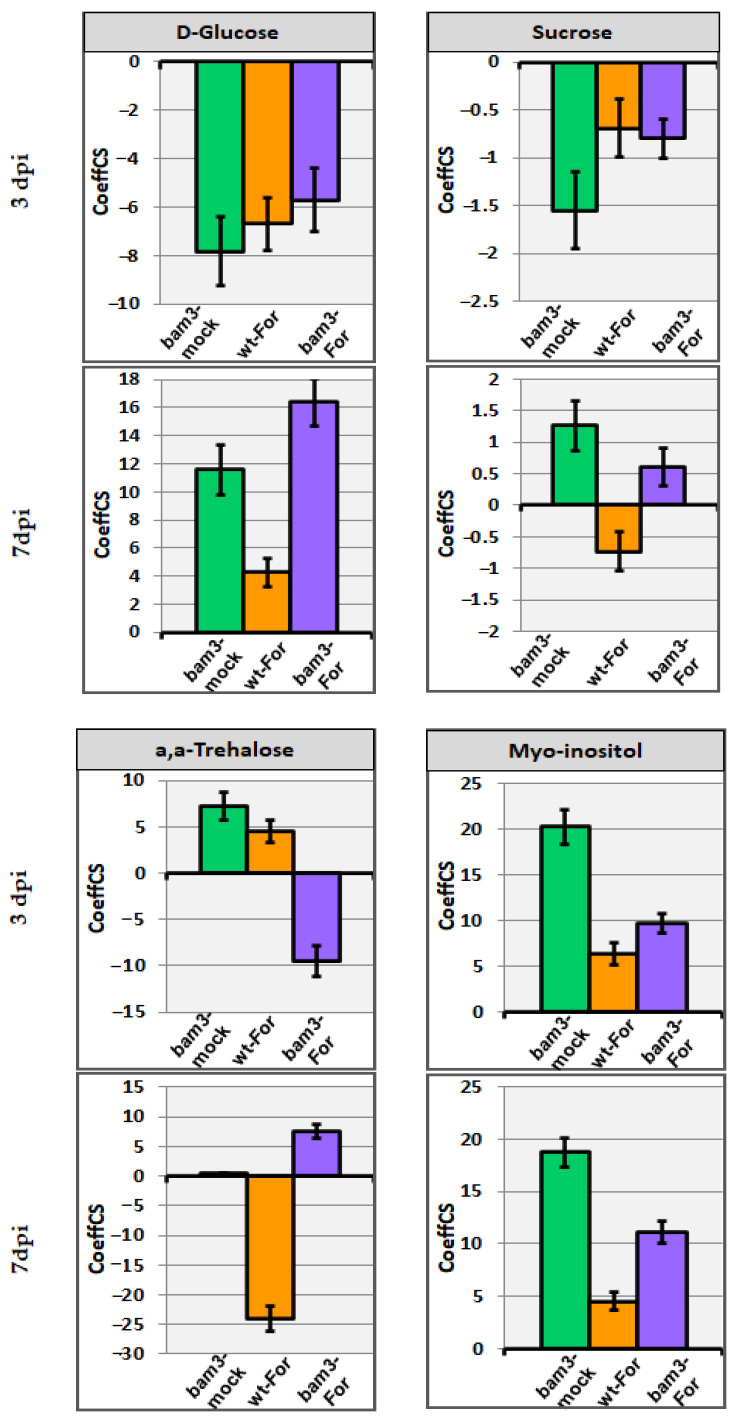
Centered partial-least squares (PLS) regression coefficients (CoeffCS) for the selected metabolites belonging to carbohydrates; D-glucose, sucrose, a,a-trehalose, and myo-inositol. CoeffCS represents the relative differences in metabolite concentrations among mock *bam3* plants, *For*-inoculated wt plants, and *For*-inoculated *bam3* plants compared to wt mock plants (x axis) at three and seven dpi, displayed with Jack-knifed confidence intervals (*p* < 0.05).

In the absence of *For*, *bam3* plants contained 7.8- and 1.6-fold less glucose and sucrose, respectively, at three dpi, while at seven dpi, glucose increased by 11.6-fold and sucrose was not differentiated (Figure 6). *For* inoculation seems to have no significant effect on sucrose production in both genotypes. On the contrary, the accumulation of glucose was reduced in both genotypes at three dpi (6.7 and 5.7-fold, in wt and *bam3* plants, respectively), but increased 4.3- and 16.4-fold in wt and *bam3* plants, respectively, at seven dpi (Figure 6).

Noninoculated *bam3* plants showed 7.2-fold higher trehalose concentrations at three dpi and no differentiation at seven dpi compared to mock wt plants (Figure 6). After inoculation with *For*, mutants produced 9.5-fold less and 7.6-fold more trehalose at three and seven dpi, respectively (Figure 6). On the contrary, the concentration of the disaccharide increased 4.6-fold and then decreased 24-fold at three and seven dpi, respectively, in *For*-inoculated wt plants (Figure 6).

Myo-inositol concentration was 20.2- and 18.8-fold higher at three and seven dpi, respectively, in mock *bam3* plants compared to the corresponding wt plants. Additionally, *For* inoculation increased the production of the carbohydrate in mutants (9.7- and 11.1-fold at three and seven dpi, respectively) (Figure 6). 

#### 2.6.2. Indole-3-Lactic Acid and 1H-Indole-3-Acetonitrile as Biomarkers

**Figure 7:**
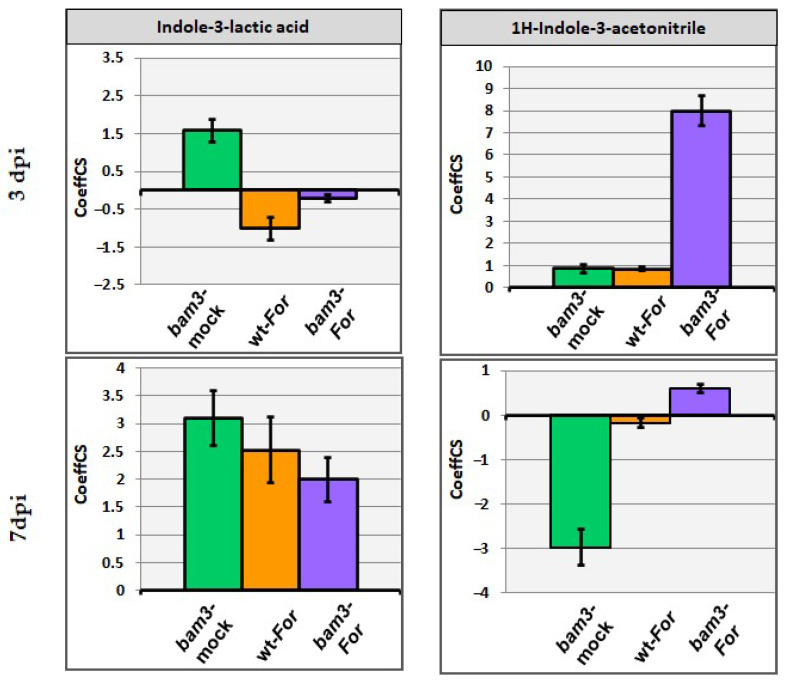
Centered partial-least squares (PLS) regression coefficients (CoeffCS) for the selected metabolites belonging to alkaloids: indole-3-lactic acid and 1H-indole-3-acetonitrile. CoeffCS represents the relative differences in metabolite concentrations among mock *bam3* plants, *For*-inoculated wt plants, and *For*-inoculated *bam3* plants compared to wt mock plants (x axis) at three and seven dpi, displayed with Jack-knifed confidence intervals (*p* < 0.05).

The Indole-3-lactic (ILA) concentration was higher in mock *bam3* plants compared to mock wt plants (1.6- and 3.1-fold at three and seven dpi, respectively), while the metabolite concentration was increased in *Fo*r-inoculated wt and *bam3* plants only at seven dpi (2.5- and 2-fold, respectively) (Figure 7).

Concerning the accumulation of 1H-Indole-3-acetonitrile (IAN), no significant alterations were observed, with the exception of an 8-fold increase in *Fo*r-inoculated *bam3* plants at three dpi and a 3-fold decrease in mock *bam3* plants at seven dpi compared to mock wt plants (Figure 7).

### 2.7. Expression of Arabidopsis Genes Related to Trehalose and Myo-Inositol Metabolism in bam3 Mutant in Response to F. oxysporum f. sp. raphani Inoculation

**Figure 8:**
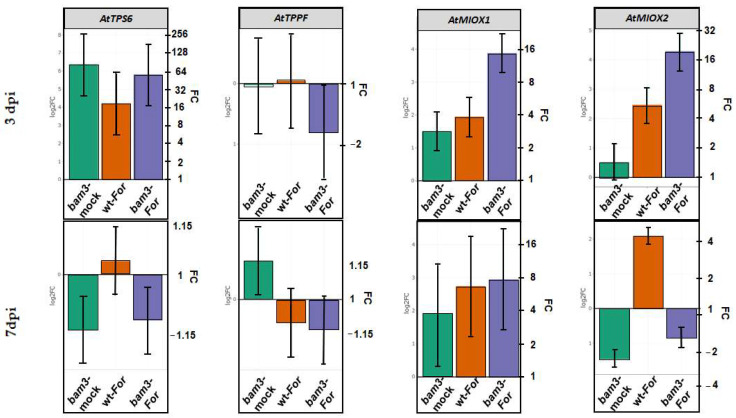
Comparative transcriptional analysis of *TPS6* and *TPPF*, related to trehalose metabolism, and *MIOX1* and *MIOX2*, related to myo-inositol metabolism. Fold change represents the relative difference in expression between each treatment (i.e., mock *bam3* plants, *For*-inoculated wt plants, and *For*-inoculated *bam3* plants) and control (i.e., wt mock plants) (x axis) at three and seven dpi. Three independent biological replicates, with two technical replicates, were analyzed, and *β-ACTIN2*, *β-ACTIN7*, and *β-ACTIN8* were used as reference genes. Bars indicate the 95% confidence interval of the estimates (2*SE) in all panels. Statistical analysis was performed with linear mixed models. Estimates for which the confidence interval includes the baseline value 0 (1 in fold-change scale) are not significantly different from control at α = 5%.

Changes in expression levels of *TPS6* (trehalose-6-phosphate synthase) and *TPP5* (CPuORF26, trehalose-6-phosphate phosphatase F) related to trehalose metabolism were determined by RT-qPCR in mock and *For*-inoculated wt and *bam3* plants at three and seven dpi (Figure 8). The above genes were chosen as differentially expressed genes (DEGs) (threshold |log_2_FC| > 1.5 and *p*-value ≤ 0.05) according to the results of preliminary microarray experiments (data not shown). *TPS6* expression was increased 83-, 18.6-, and 56.4-fold in mock *bam3* plants, *For*-inoculated wt plants, and *For*-inoculated *bam3* plants, respectively, compared to wt mock plants. *TPS6* expression reduced 4.5-fold in *For*-inoculated *bam3* plants compared to mock mutant plants at three dpi (Figure 8). On the contrary, at seven dpi, relative transcript levels of the gene were very low in both genotypes. RT-qPCR expression of *TPP5* was not altered in mock *bam3* and *For*-inoculated wt plants, but increased in *For*-inoculated *bam3* plants at three dpi (Figure 8). At seven dpi, relative transcript levels of the gene were higher in mock *bam3* plants compared to wt plants (1.1-fold), while *For* inoculation did not affect the gene expression in both genotypes (Figure 8).

Concerning myo-inositol metabolism, the *MIOX1* and *MIOX2* (myo-inositol oxygenase 1 and 2) genes were chosen for expression analysis according to the results of preliminary microarray experiments (data not shown). Inoculation with *For* induced the relative levels of *MIOX1* and *MIOX2* mRNAs in both genotypes at three dpi, but the induction was higher in mutants compared to mock wt (3.9- and 5.5-fold in wt, 14.7- and 19.6-fold in *bam3*, respectively, for the two genes). Additionally, the transcript levels of the genes were almost 4-fold higher in inoculated *bam3* mutants compared to inoculated wt plants (Figure 8). The transcript levels of *MIOX1* were induced but not significantly differentiated among biological groups at seven dpi. In contrast, *MIOX2* was downregulated in mock and *For-*inoculated mutants at seven dpi (2.8- and 1.8-fold, respectively) (Figure 8).

### 2.8. Expression of GH3.3 and DRM2 Genes Related to Auxin Metabolism in bam3 Mutants upon Inoculation with F. oxysporum f. sp. raphani

**Figure 9:**
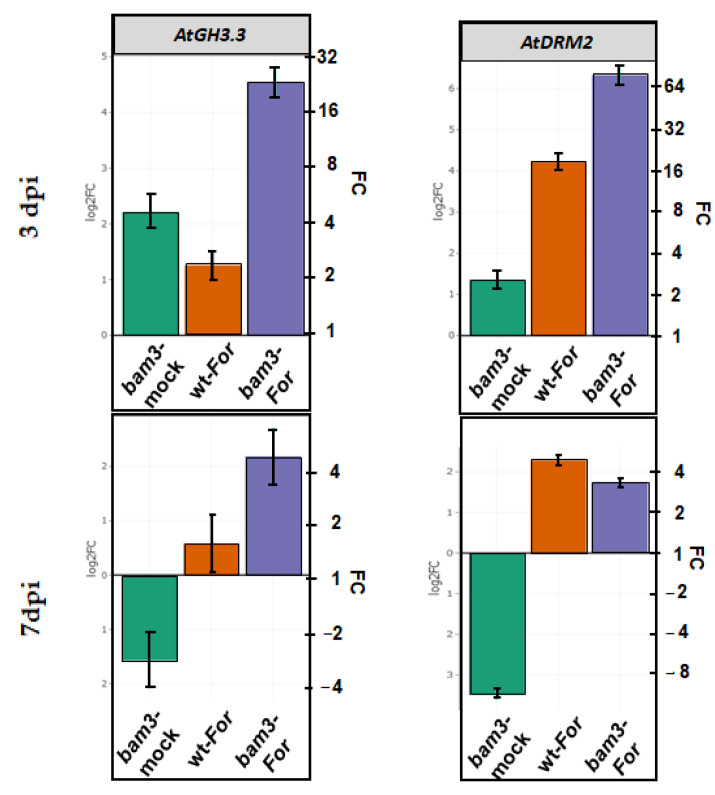
Comparative transcriptional analysis of GH3.3 and DRM2 related to auxin metabolism. Fold change represents the relative difference in expression between each treatment (i.e., mock bam3 plants, For-inoculated wt plants, and For-inoculated bam3 plants) and the control (i.e., wt mock plants) (x axis) at three and seven dpi. Three independent biological replicates, with two technical replicates, were analyzed, and β-ACTIN2, β-ACTIN7, and β-ACTIN8 were used as reference genes. Bars indicate the 95% confidence interval of the estimates (2*SE) in all panels. Statistical analysis was performed with linear mixed models. Estimates for which the confidence interval includes the baseline value 0 (1 in fold-change scale) are not significantly different from control at α = 5%.

*GH3.3* and *DRM2* (AT2G33830) related to auxin metabolism were chosen for the expression analysis according to the results of preliminary microarray experiments (data not shown). In the present study, *GH3.3* was up- and downregulated at three and seven dpi, respectively, in mock *bam3* plants compared to mock wt plants (4.6- and 3-fold) (Figure 9). *For* inoculation led to 23.2- and 4.5-fold increases in *GH3.3* expression in inoculated *bam3* plants at three and seven dpi, respectively (Figure 9).

In the absence of the pathogen, *bam3* plants exhibited a 2.6-fold increase and an 11.3-fold decrease in *DRM2* expression at three and seven dpi, respectively (Figure 9). After *For* inoculation, *DRM2* was overexpressed in both genotypes, but the gene induction was significantly greater in *bam3* plants (80.2- and 3.4-fold in *bam3*, and 18.5- and 4.9-fold in wt, at three and seven dpi, respectively) (Figure 9).

## 3. Discussion

In the present study, *A. thaliana bam3* plants exhibited lower disease severity and had limited fungal DNA amounts in its vascular system upon inoculation with *For*. According to Gkizi et al. [20], Arabidopsis *bam3* plants showed less Verticillium-wilt symptoms and lower *V. dahliae* colonization levels compared to wt plants. Therefore, BAM3 seems to be a negative regulator of disease resistance to host-specific strains of *Fo*, as well as to *Verticillium dahliae* [20], both of which are soil-borne pathogens that commonly cause vascular-wilt diseases, leading to economically important losses in a wide range of crops. Similarly, Perlikowski et al. [21] found that plant *β*-amylase activities are involved in plant–pathogen interactions associated with the resistance to *Fusarium culmorum*, causing Fusarium head blight (FHB) in wheat.

Bam3 is the most catalytic protein required for starch breakdown, since *bam3* mutants have elevated starch levels and lower night-time maltose levels compared to wt plants [9]. Consistent with the above, our results showed that mock *bam3* plants exhibited significantly lower expressions of *BAM3* and contained less maltose than wt plants at three and seven dpi.

*For* inoculation caused no statistically significant fluctuations in maltose and starch concentrations in wt and *bam3* plants at three and seven dpi. The pathogen affected the expression of genes related to starch degradation at three dpi, but not at seven dpi, with the exception of *BAM3.* Several of the genes involved in starch degradation showed diurnal changes in expression. Despite the evidence for strong diurnal/circadian regulation of their transcription, the levels of the encoded proteins (when determined) generally did not fluctuate in the same way as the transcript, and instead remained relatively constant [6]. Additionally, changes in sugar levels or other metabolic intermediates may also serve as the trigger for the fluxes into and out of the starch pool [6]. Therefore, environmental and endogenous conditions, such as light intensity, period and quality, temperature, developmental stage, sugar status, biotic and abiotic stresses, etc., can cause changes in allocation into and out of the starch pool [6].

*BAM1*, *AMY3*, *ISA3*, and *DPE1* were overexpressed in *bam3* plants, while the expression of *BAM3* was reduced in the presence and absence of *For* at the early stages of infection, i.e., at three dpi. *BAM2* and *BAM4,* encoding plastid-localized enzymes with activity far lower than that of BAM3 [9], were not altered in *bam3* plants upon *For* inoculation. According to Horrer et al. [22], BAM1 does not seem to affect starch metabolism in mesophyll cells under normal conditions, but only upon illumination, during stomatal opening during the day, and in guard cells in conjunction with the chloroplastic a-amylase3 (AMY3), leading to glucose as the major starch-derived metabolite in Arabidopsis guard cells [23]. Gamir et al. [24] observed that infection with the fungus *Plectosphaerella cucumerina* induced the expression of the *BAM1* gene while decreasing the transcript levels of *BAM3*. For the complete degradation of starch, activity of the phosphoglucan phosphatase starch excess 4 (SEX4) and the debranching enzyme isoamylase 3 (ISA3) are required [5]. Subsequently, the enzyme disproportionating enzyme 1 (DPE1) leads to the production of glucose [25].

**Figure 10:**
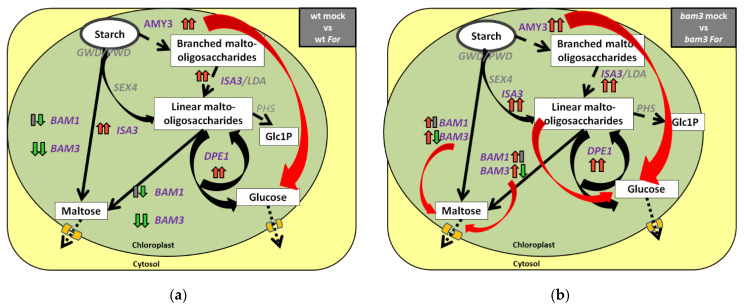
Transcriptional regulation of starch degradation in chloroplasts of *Arabidopsis thaliana* (**a**) wt plants and (**b**) *bam3* mutants 3 days (left arrow) and 7 days (right arrow) post inoculation (dpi) with *Fusarium oxysporum* f. sp. *raphani* (*For*). Genes studied are written in purple, while red and green arrows indicate up- and downregulated genes, respectively, while grey rectangles indicate no significant differentiation. Estimated fluxes are indicated by relative arrow size. Dashed arrows represent the minor steps in Arabidopsis. Initially, glucan water dikinase (GWD) and phosphoglucan water dikinase (PWD) disrupt the packing of amylopectin double helices by phosphorylation, while α-amylases (AMY3) seems to help degradation as a minor step, releasing branched maltooligosaccharides. Then, β-amylases (BAM1 and BAM3) and the debranching enzyme (DBE) isoamylase 3 (ISA3) release maltose and maltooligosaccharides (black arrows). Phosphoglucan phosphatases, such as SEX4 and the DBE (ISA3), complete the degradation. Short maltooligosaccharides are also metabolized by the glucanotransferase disproportionating enzyme (DPE1) producing glucose and a spectrum of linear oligosaccharides, the larger of which are again substrates for the other degradative enzymes. Maltooligosaccharides are metabolized in the stroma, while maltose and glucose are exported to the cytosol. In the present study, transcriptional regulation of *BAM1*, *BAM3*, *ISA3*, *AMY3*, and *DPE1* leads towards more glucose production in *A. thaliana bam3* plants compared to wt, especially after inoculation with *For* (b, big red arrows).

The transcriptional regulation of the above genes of starch metabolism leads mainly towards glucose production in *bam3* plants, a regulation that is higher, especially after *For* inoculation (Figure 10), indicating a possible involvement in Arabidopsis *bam3* mutant defense against the pathogen. Supporting evidence to the above-mentioned are our results of the metabolomic analysis, according to which the mock and *For*-inoculated *bam3* plants contained more D-glucose compared to mock wt plants at seven dpi. Sugars constitute the primary substrate providing energy, as well as the structural material for defense responses in plants. They may also act as signal molecules, interacting with the hormonal signaling network regulating the plant immune system [26]. Formela-Luboińska et al. [12] revealed the involvement of sucrose, glucose, and fructose as primary signals in the regulation of the level of signaling molecules, such as phytohormones, SA, jasmonates (JA and MeJA), ET, ABA, and H_2_O_2_, and NO during the defense response of *Lupinus luteus* against the hemibiotrophic fungus *F. oxysporum* f. sp. *lupini*. It is well known that glucose is phosphorylated by a hexokinase (HXK) to glucose 6-phosphate (G6P) and added to the reserves of hexoses to subsequently be used to produce sucrose, the disaccharide trehalose, and/or inositol 1-phosphate [26]. Many studies have also shown that glucose activates the expression of several PR genes, and that glucose sensor hexokinase (HXK1) and G-protein-coupled receptor (RGS1) are involved in defense responses [27].

Trehalose synthesis is a two-step process, involving the production of trehalose-6-phosphate (T6P) catalyzed by trehalose-6-phosphate synthase (TPS) and its consecutive dephosphorylation to trehalose, catalyzed by trehalose-6-phosphate phosphatase (TPP) [13]. In our study, transcript levels of *TPS6* were higher in mock and *For*-inoculated *bam3* plants, while the expression of *TPPF* was reduced in mock and *For*-inoculated mutants at three dpi. On the contrary, at seven dpi, the pathogen did not seem to significantly affect the expression of the above genes in either genotype. Additionally, metabolomic analysis revealed that mock *bam3* plants contained more trehalose compared to wt, which seems to be in line with the expression analysis of the above genes. However, *For* inoculation caused a significant decrease in this metabolite concentration in mutants at three dpi, maybe because of the observed reduction in *TPPF,* suggesting a possible increase in T6P accumulation. To date, most research work has focused on the role of *TPS1* encoding an active TPS proteins [28], while AtTPS6 appears to be an active trehalose synthase that plays an important role only in the growth and control of cell morphogenesis [29]. According to our results, transcriptional regulation of *AtTPS6* appears to play an important role in trehalose concentrations in *bam3* plants, and maybe in their defense response against *For*. This is because trehalose and its precursor, T6P, are considered to be important signaling molecules, regulating defense responses through complex sugar-sensing pathways.

There is sufficient evidence to suggest that sucrose, glucose, and T6P act as signals, reflecting the general metabolic health of plants and also acting as regulators of plant defense in some cases [30,31,32]. In our study, *bam3* plants with a decrease in maltose production showed an increase in glucose, sucrose, and trehalose concentrations seven days after inoculation with *For*. This suggests that the pathogen may regulate the transient breakdown of starch and balance the supply of substrates for sucrose synthesis, leading to the regulation of sugar fluxes that, in turn, induces defense responses against the Fusarium-wilt pathogen at the early stages of infection. Actually, the resistant phenotype of *A. thaliana bam3* plants is related to SA-JA/ET-mediated pathways (Kalogeropoulou, unpublished data).

Metabolite data also revealed that myo-inositol, a carbohydrate produced by most plants, was induced in mock and *For*-inoculated *bam3* plants compared to wt. Myo-inositol (MI) is a small molecule that is important in many different developmental and physiological processes in eukaryotic cells. It participates in the phosphatidylinositol (PtdIns) signaling pathway, auxin storage and transport, phytic acid biosynthesis, cell-wall biosynthesis, and the production of stress-related molecules [33]. Its metabolism is also implicated in plant programmed cell death (PCD), in the generation of reactive oxygen species (ROS), such as H_2_O_2_, and in the regulation of defense-related gene expression [34,35,36].

Transcriptomic analysis revealed that inoculation with *For* caused higher inductions of *MIOX1* and *MIOX2* expressions in *bam3* plants, enhancing the myo-inositol oxidative pathway in mutants upon *For* inoculation. The MI oxidative pathway effectively consumes MI and might be activated under sugar-starvation conditions to generate alternative sugar sources, thereby indirectly contributing to metabolic homeostasis [33]. In addition, MIOX is a unique monooxygenase that catalyzes the conversion of myo-inositol to D-glucuronic acid (D-GlcUA), which ultimately enters the pool of UDP-GlcA and serves as a precursor for the polysaccharides (mainly pectin) of plant cell walls [33]. This is in line with the most recent findings related to the role of cell-wall pectin content and structure in *bam3* defense responses against *For* (Kalogeropoulou, unpublished data).

Together with other phytohormones, IAA is responsible for plant growth and development, and is involved in nearly every plant process, such as cell growth, root initiation, tropism, fruit ripening, or senescence [37]. Additionally, it plays a role in the communication between host plants and plant pathogens [37]. In our study, the precursor of indole-3-acetic acid (IAA), indole-3-lactic acid (ILA), had higher concentrations in mock and *For*-inoculated *bam3* plants compared to mock wt plants at seven dpi. In addition, transcriptomic analysis revealed that *For* induced the expression of *GH3.3* and *DRM2* genes in *bam3* plants, an induction that was particularly noticeable at three dpi. *GH3* genes encode plant-specific proteins with a role in hormone conjugation, acting on auxins, jasmonic acid (JA), salicylic acid (SA), and benzoates [38]. Although most GH3s exist as conjugating enzymes, some GH3 family members can play a role in plant responses to both biotic and abiotic stress conditions [39]. Specifically, *GH3.3* encodes the protein that conjugates aspartic acid (Asp), and the arising conjugate has in turn been reported to play a key role in the regulation of defense against *Botrytis cinerea* and *Pseudomonas syringae* pv. *tomato DC3000*, since it was found that GH3 proteins can induce defense responses (e.g., SA) in Arabidopsis, tomato (*Solanum lycopersicum*) and tobacco plants (*Nicotiana benthamiana*) after infection with the above pathogens [15]. Concerning *DRM2* (or *DAP2*), encoding DORMANCY/AUXIN ASSOCIATED FAMILY PROTEIN 2, there is evidence that DRM1 and -2 proteins are associated with defense responses against biotic and abiotic stresses [40]. Contrary to our results, Roy et al. [41] reported that DRM2 expression was upregulated after bacterial infection, causing the suppression of the defenses against the bacterial strains, pathogen-induced callose deposition, and ROS accumulation. Therefore, further research should be conducted in order to investigate the role of DRM2 protein in plant defense against fungi.

In the past, it has been reported that some genetic modifications in auxin transport and signaling caused induction of defense against *F. oxysporum* [42]. Such modifications affect indirectly the regulation of cell-wall architecture, root morphology, and stomatal development and patterning [15,43], or induce directly plant defenses through the mechanisms of pathogen-triggered immunity [44]. Apart from that, Mishra et al. [45] reported that auxin signaling affects sugar metabolism and carbon partitioning. Thus, further research should be conducted in order to investigate the relationship between these two signaling pathways in the *A. thaliana bam3* mutant upon inoculation with *For*.

## 4. Materials and Methods

### 4.1. Biological Material and Bioassays

In this study, seeds of *Arabidopsis thaliana* ecotype Col-0 (wt) and its mutant *bam3*, provided by S. C. Zeeman (Institute of Plant Sciences, ETH Zurich, Switzerland), were used. Until their use, they were maintained at 4 °C. *Fusarium oxysporum* f. sp. *raphani (For)* isolate WCS600 (provided by C. M. J. Pieterse; Utrecht University, The Netherlands), with known pathogenicity against *A. thaliana* plants [46,47], was used. The fungal isolate was cryopreserved at −80 °C by freezing a conidial suspension in 25% aqueous glycerol [47]. Before use, the fungus was transferred to potato dextrose agar (Merck, Darmstadt, Germany) and incubated at 27 °C for five days.

The bioassays were conducted in a controlled environment growth room following a completely randomized design. Seeds were sown in plastic pots (9 cm × 9 cm × 10 cm) containing sterile compost (Potground; Klasmann), incubated for three days at 4 °C for dormancy breaking, and then maintained in a controlled environment growth room at 25 °C with a 16 h photoperiod for germination. Twelve days post sowing, when the first pair of true leaves appeared, plants were transplanted individually into plastic pots (7 cm × 7 cm × 6 cm) containing approximately 150 cm^3^ of sterile compost. They were grown at 25 °C with a 16 h light (130 μmol m^−2^ s^−1^)/8 h dark photoperiod and were watered twice a week by reverse watering. Three-week-old plants were inoculated with *For* by root drenching with 12 mL of a suspension containing 10^6^ conidia mL^−1^ in sterile distilled water (SDW) [47]. The suspension of 10^6^ conidia mL^−1^ of SDW was prepared from a culture grown for three days in sucrose sodium nitrate liquid medium at 27 °C [47]. Control plants were mock-inoculated with 12 mL of SDW (mock plants). The bioassay consisted of 30 plants per genotype, treatment, and time point.

For metabolome and transcriptome analyses and measurement of the carbohydrate content, the above soil parts (leaves and stem tissues) of the *For*-inoculated and mock wt and *bam3* plants of the same bioassay were collected at three and seven dpi, at the end of the of the dark period, flash-frozen in liquid nitrogen, and stored at −80 °C until further use.

### 4.2. Disease Assessment

The number of leaves that showed wilting as a percentage of the total number of leaves present on each plant was periodically recorded for 24 days post inoculation (dpi), starting at 11 dpi. Disease ratings were plotted over time to generate disease-progress curves. The area under the disease-progress curve (AUDPC) was calculated by the trapezoidal integration method [48]. Disease severity was expressed as a percentage of the maximum possible AUDPC for the whole period of the experiment and is referred to as the relative AUDPC. Data were analyzed using the SGWIN software (STATGRAPHICS Plus version 2.1, Statgraphics Technologies, Inc., Virginia, USA) and by applying analysis of variance (ANOVA). When a significant (*p* ≤ 0.05) *F*-test was obtained for treatments, data were subjected to means separation by Fisher’s LSD multiple-range test (*p* ≤ 0.05).

### 4.3. Measurement of For Quantity in Inoculated Plants

For fungal biomass estimations, real-time PCR analysis was performed. Initially, from each *A. thaliana* genotype (wt and *bam3* mutant), treatment (mock- and *For-*inoculated plants) and time point (12 and 24 dpi), the above soil parts (leaves and stem tissues) of 10 plants were harvested and pooled, resulting in one biological sample. Three independent bioassays were conducted, with each bioassay consisting of 30 plants per Arabidopsis genotype and treatment (total of 90 plants). For each sample, the above-ground plant parts (leaves and stem tissues) were flash-frozen and pulverized under liquid nitrogen. Total DNA was extracted according to Dellaporta et al. [49] and was quantified by NanoDrop^®^ ND-1000 Spectrophotometer. The qPCR assays for *For* quantification were conducted using the primer pair PG5-F and PG5-R (Supplementary Table S1) designed for the endopolygalacturonase *PG5* gene (AB256876) of *F. oxysporum* [47]. KAPA SYBR^®^ FAST qPCR Master Mix (2X) Universal (Kapa Biosystems, Wilmington, MA, USA) was used and qPCR reactions were performed in an Applied Βiosystems StepOnePlus Real-Time PCR thermocycler using the following conditions: initial denaturation at 95 °C for 3 min, followed by 40 cycles, each consisting of 3 s at 95 °C and 30 s at 60 °C. After each run, a dissociation curve was acquired by heating the samples from 60 °C to 95 °C to check for amplification specificity and formation of primer dimers. The results were analyzed with StepOne v.2.3. software (Applied Biosystems, Thermo Fisher Scientific Inc.). The *A. thaliana a2-tubulin* gene (M84696) was used as a reference gene (primers listed in Supplementary Table S1) [47,50,51]. All reactions were performed in duplicates. Data were analyzed using the SGWIN software (STATGRAPHICS Plus version 2.1, Statgraphics Technologies, Inc., Virginia, USA) and applying analysis of variance (ANOVA). When a significant (*p* ≤ 0.05) F-test was obtained for treatments, data were subjected to means separation by Fisher’s LSD multiple-range test (*p* ≤ 0.05).

### 4.4. Sampling and Metabolite Extraction

Metabolomic analysis was performed with gas chromatography-mass spectrometry (GC/MS) in the above-soil parts (leaves and stem tissues) of the *For*-inoculated and mock wt and *bam3* plants collected at three and seven dpi. Each biological sample consisted of six randomly selected plants per sampling day, genotype, and treatment (wt and *bam3* plants either *For*- or mock-inoculated). Each sample was flash-frozen, pulverized under liquid nitrogen and an amount of 40 mg was weighted into 2.0 mL Eppendorf tubes and stored at −80 °C until further processing. Five independent biological samples were analyzed. Metabolite extraction was performed as previously described [52]. Briefly, 1 mL of a mixture of methanol:ethyl acetate (50:50, *v*/*v*) was added to 40 mg of frozen plant tissue of each biological sample followed by sonication in an ultrasonic bath (Branson 1210, Connecticut, NE, USA) for 20 min. Samples were spiked by adding 20 µL of a ribitol solution (0.2 mg mL^−1^) in methanol:water (50:50, *v*/*v*) and transferred to an orbital shaker (GFL 3006, Gesellschaft für Labortechnik mbH, Burfwedel, Germany) for extraction under continuous agitation in 200 rpm for 2 h at room temperature. Extracts were filtered through 0.25 mm filters (Macherey-Nagel, Duren, Germany) to remove debris and the volume of sample was adjusted to 1.0 mL. Finally, extracts were dried overnight using a centrifugal evaporator (Labconco Corporation, Kansas City, MO, USA) at 45 °C.

### 4.5. Chemical Analyses and Data Processing

Derivatization of samples was performed as previously described [53]. Briefly, 80 µL methoxylamine hydrochloride (Sigma-Aldrich, St. Louis, MO, USA) in pyridine (Sigma-Aldrich) (20 mg mL^−1^) was added to the dried extracts followed by incubation at 30 °C for 120 min. In a second step, 80 µL MSTFA (Sigma-Aldrich) was added, and samples were incubated at 37 °C for 90 min. The derivatized samples (150 µL) were transferred into microinserters (Fisher Scientific Company, Hampton, VA, USA) placed in glass autosampler vials (2 mL) and analyzed with an Agilent 7890A gas chromatograph platform (Agilent Technologies Inc., Santa Clara, CA, USA) equipped with a 7683 series autosampler and coupled with a 5973 inert mass selective detector (MSD). The electron ionization was set at 70 eV and full scan mass spectra were acquired at the mass range of 50–800 Da at rate 2 scan/s with a 10 min solvent delay. The temperature for the ion source was set to 150 °C, for the transfer line to 230 °C, and for the injector to 230 °C. Samples (1 µL) were injected using a split ratio of 10:1 into a HP-5MS ultra inert (UI) capillary column (30 m × 250 µm I.D., 0.25 µm film thickness; Agilent Technologies Inc.). The initial temperature of the oven was 70 °C stable for 5 min, followed by a 5 °C min^−1^ increase to 295 °C and finally stable for 2 min. Helium was used as the carrier gas at a constant flow rate 1 mL min^−1^. Calibration of the instrument was performed daily throughout the course of the analyses using the default automatic calibration mode as recommended by the manufacturer. Chemicals and reagents used were of the highest purity grade commercially available.

Chromatogram acquisition, peak deconvolution, and MS library searches were performed using the Agilent MSD Chemstation version E.02.00.493. Automatic integration was performed after optimization of the ChemStation Integrator parameters based on the total ion chromatogram (TIC). Putative identification of metabolites was performed by matching their mass spectra to spectra in NIST 08 library (National Institute of Standards and Technology, Gaithersburg, MD, USA). The definitive identification of metabolites was based on matching their mass spectra and retention times (RT) to those of the authentic chemical standards analyzed on the same platform with the same analytical method. Tentative identification was performed for metabolites with a very good fit (>90%). Metabolites of nonbiological origin were excluded from further analysis. The above data and those obtained from Golm Metabolom Database (GMD, Version: 18.10.5, http://gmd.mpimp-golm.mpg.de/) (accessed on 15 June 2018), Kyoto Encyclopedia of Genes and Genomes (KEGG, https://www.genome.jp/kegg/) (accessed on 15 June 2018) and PubChem (https://pubchem.ncbi.nlm.nih.gov/) (accessed on 15 June 2018) were exported as ‘‘.txt’’ files to MS Excel^®^ for the creation of data matrices.

### 4.6. Quality Control of Metabolomic Analyses

Standard operating procedures (SOP) and quality control (QC) measures were followed throughout the experimental steps to ensure the quality and validity of the analyses. For each treatment, a QC sample was obtained by pooling aliquots of the five biological replications. Additionally, blank samples were processed alongside the experimental samples and were subjected to identical handling for the detection of possible sources of contamination during the different experimental steps, such as contamination during sample preparation, column bleeding, solvent impurities, or instrument contamination. Furthermore, technical replications of randomly selected samples were performed to access the reproducibility of analytical conditions. Samples were analyzed in completely randomized order to avoid possible variability caused by inconsistent performance of the analyzers.

To maintain instruments’ performance, calibration of the analyzers was performed following the recommended manufacturers’ procedures and using calibration solutions. Tuning of the MS detector was performed automatically using the AutoTune function and the DRO/GRO Range Calibration Standard was injected every 10 samples to monitor the performance of the instrument. Additionally, samples were spiked with ribitol to monitor possible shifts in retention time and the reproducibility of analyses.

### 4.7. Statistical Analyses

Data matrices were subjected to multivariate analyses using the SIMCA-P+ v.12.0 software (Umetrics, MKS Instruments Inc., Andover, MA, USA) for the detection of trends and corresponding metabolite-biomarkers as previously described [52,53]. Initially, principal component analysis (PCA) was performed for the evaluation of data and detection of outliers.

The discovery of biomarkers was based on scaled and centered partial-least square-discriminant analysis (PLS-DA) regression coefficients (*p* < 0.05), since by applying PCA, it is not certain that the computed principal components (PCs) represent the largest sources of variation [54]. Standard errors were calculated using Jack-knifing (95% confidence interval). The performance of the obtained models was assessed by the cumulative fraction of the total variation of the X’s that could be predicted by the extracted components [Q2 (cum)] and the fraction of the sum of squares of all X’s (R2 X) and Y’s (R2 Y) explained by the current component. Additionally, data were subjected to One-Way ANOVA performing the Student’s *t*-test (*p* < 0.05) using the software JMP8.0 (SAS Institute Inc., Kari, NC, USA).

### 4.8. RNA Isolation and Gene-Expression Analysis

The gene-expression analysis was performed with reverse-transcription quantitative PCR (RT-qPCR). Three independent biological samples were analyzed. Each biological sample consisted of 10 randomly selected plants per sampling day (3 and 7 dpi), genotype (wt and *bam3* plants) and treatment (*For*- or mock-inoculated). Plant tissues were flash-frozen and pulverized under liquid nitrogen and stored at −80 °C. Total RNA was isolated using NucleoSpin^®^ RNA Plant Kit (Macherey-Nagel GmbH & Co., Düren, Germany) according to the manufacturer’s instructions. It was quantified using a NanoDrop^®^ ND-1000 Spectrophotometer while its quality was confirmed by agarose gel electrophoresis. cDNA was synthesized using QuantiTect^®^ Reverse Transcription kit (Qiagen) according to the manufacturer’s instructions. Quantitative RT-PCRs were performed on the Applied Biosystems StepOnePlus Real-Time PCR thermocycler using the master mix SYBR^®^ Select Master Mix for real-time PCR (SYBR GreenER™ dye) (Applied Biosystems, Thermo Fisher Scientific Inc.). Each reaction contained 5 μL of SYBR^®^ Select Master Mix (2X) (Applied Biosystems, Thermo Fisher Scientific Inc.), 0.4 μL of 10 μM of each gene-specific primer pair and 1 μL of cDNA template to a final volume of 10 μL.

Candidate genes were selected based on their differentially expression according to the results of microarrays preliminary experiments (data not shown) and their homology to genes known to play a role in sugar metabolism and in β-amylolysis. All primers used are described in Supplementary Table S1 and were tested with Primer Express™ Software v3.0.1 (https://www.thermofisher.com/order/catalog/product/4363993, Applied Biosystems) (accessed on 20 Mar. 2015). All reactions were performed in duplicate. The absence of nonspecific products and primer dimers was confirmed by the analysis of melting curves. In all cases, the Arabidopsis housekeeping genes *β-ACTIN2* (AT3G18780), *β-ACTIN7* (AT5G09810) and *β-ACTIN8* (AT1G49240) were used as reference genes [55,56,57,58]. qPCR data were analyzed within a Linear Mixed Model (LMM) framework (R: lmer package [59]). Ct values comprised the dependent variable, while gene, treatment (infection type) and their interaction formed the fixed components of the LMM. Sample IDs constituted the random part of the model. Contrasts of estimated values in log2 scale were accordingly formulated as expressions of relevant ΔΔCts [60] according to Equation 5 of ref. [61], by implementing the emmeans [62] and limma [63] packages in R [64]. In cases where calibration between runs was needed due to large number of preps, calibrator samples (i.e., identical sample preps) were included in every plate to make possible the estimation of calibration factors. Calibration factors (CFs) were estimated before the main analysis using the same methodology. After the correction of Ct values according to CFs, calibrator samples were excluded from the main analysis.

### 4.9. Quantification of Maltose and Starch-Derived Glucose Contents

For estimation maltose and starch-derived glucose contents in *A. thaliana* wt and *bam3* plants 3 and 7 days after *For*- or mock-inoculation, a photometric assay was used according to Smirnova et al. [65]. The biological samples of the assay were the same as those used for the gene-expression analysis. Initially, 50 mg of ground, frozen tissue of each biological sample comprising a mixture of 10 plants were mixed with 1 mL 80% (*v*/*v*) ethanol each followed by vigorous mixing and heated at 80 °C for 15 min. After centrifugation (10 min at 20,000× *g*; room temperature), the supernatant was collected while the pellet was re-extracted resuspended in 1 mL fresh 80% (*v*/*v*) ethanol as described before. The combined two supernatants, referred to as heat-stable low molecular weight compounds, were used for maltose quantification while the pellet was processed for starch determination (see below). The content of buffer-soluble proteins was quantified using a NanoDrop^®^ ND-1000 Spectrophotometer.

Assay for maltose quantification was based on the activity of maltase from yeast (a-glucosidase; E.C. 3.2.1.20, Megazyme). A two-step assay was performed using a 96-well microplate according to Smirnova et al. [65]. Primarily, 100 mM citrate-NaOH pH 6.5 and 2 U maltase were added in supernatants (heat-stable low molecular weight compounds in a final volume of 100 μL. After incubation for 30 min, absorbance at 340 nm was measured. Subsequently, a total of 100 μL was added containing 100 mM Tricine-NaOH pH 7.8, 5 mM MgCl_2_, 1 mM ATP (Adenosine 5′-Triphosphate Disodium Salt Hydrate > 98%, A0157, TCI), 1 mM NADP^+^ (β-Nicotinamide adenine dinucleotide phosphate sodium salt hydrate, 97+%, ACROS Organics™, AC227570000, Fisher Scientific), 0.5 U hexokinase (Hexokinase, Yeast, 376811, MERC), and 0.25 U glucose6-phosphate dehydrogenase (Glucose-6-phosphate Dehydrogenase, 346774, MERC). After 10 min, absorbance at 340 nm was measured again. A calibration curve constructed from ethanol maltose standard solutions (0–50 nM).

For starch quantification, the pellet obtained above was resuspended in 1ml cold water and after centrifugation (10 min at 20,000× *g*; room temperature), the supernatant was discharged, and the pellet was dried at room temperature (almost five hours). Subsequently, the pellet was homogenized in 0.5 mL 200 mM KOH and incubated for 1 h at 95 °C. After cooling, the sample was neutralized by adding 88 μL of 1 M acetic acid followed by centrifugation (10 min at 20,000× *g*; room temperature). An aliquot of 50 μL of the supernatant containing the solubilized starch was mixed with an equal volume of a commercial amyloglucosidase preparation (Amyloglucosidase, *Aspergillus niger*, EC: 3.2.1.3, Megazyme) and the mixture was incubated overnight at 55 °C. The starch-derived glucose concentration was quantified enzymatically using a microplate reader and a 96-well microplate (340 nm, 37 °C). For each sample, in a volume of 190 μL, the mixture contained approximately 100 mM Tricine-NaOH pH 7.8, 5 mM MgCl_2_, 1 mM ATP (Adenosine 5′-Triphosphate Disodium Salt Hydrate > 98%, A0157, TCI), 1 mM NADP^+^ (β-Nicotinamide adenine dinucleotide phosphate sodium salt hydrate, 97+%, ACROS Organics™, AC227570000, Fisher Scientific) and 0.28 U glucose-6-phosphate dehydrogenase from yeast (Glucose-6-phosphate Dehydrogenase, 346774, MERC). All the above concentrations were calculated for a final volume of 200 μL. The reaction mixture was preincubated for 10 min and absorbance at 340 nm was measured. Reaction was started by adding 0.56 U hexokinase from yeast (Hexokinase, Yeast, 376811, MERC) and was completed in less than 10 min and the absorbance at 340 nm was measured again. Starch solutions in KOH of known concentrations (0–50 nM) were used as standard samples for the construction of the standard curve.

## 5. Conclusions

Our study provides significant insights into the role of BAM3 as a negative regulator of disease resistance against *For. Bam3* plants developed a complex array of defense mechanisms against *For* by reprogramming the metabolism and gene expression related to starch degradation, regulatory function of sucrose and glucose, trehalose metabolism, myo-inositol and its oxidative pathway, the auxin biosynthesis pathway, and auxin conjugate synthesis. Based on these conclusions gained from Arabidopsis, it will be possible to move forward with research and initiate improvements in plants of economic importance, offering new approaches for breeding Fusarium-resistant crops by targeting the starch-degrading enzymes.

## Data Availability

Data are contained within the article or Supplementary Materials.

## Supplementary Materials

The following supporting information can be downloaded at: shttps://www.mdpi.com/article/10.3390/plants11243457/s1. All primers used are described in Supplementary Table S1: List of genes and primers used in this study [47,51,55,56,57,58,66,67,68,69,70,71,72,73]. Metabolomics data are illustrated in supplementary file.
