# Supplementary material for: Combined Transcriptomic and Metabolomic Analysis Reveals Insights into Resistance of Arabidopsis bam3 Mutant against the Phytopathogenic Fungus Fusarium oxysporum
Source: Plants (Basel). 2022 Dec 9;11(24):3457. doi: 10.3390/plants11243457 (PMC9785915; doi:10.3390/plants11243457)
Supplement: Supplementary file 1 [file plants-11-03457-s001.zip › Supplementary Table S1.pdf]

Table S1. List of genes and primers used in this study

Gene target	AGI locus code	Sequence (5'–3')	Reference
**Primers used for quantification *of F. oxysporum* f. sp. *raphani* by qPCR**			
PG5 (Fusarium oxysporum)	AB256876	F: 5'-CGAGGGTAAGAGATGGTGGGATGG-3'	[47]
		R: 5'-GCCGCCGGTGAAGGTGATGT-3'	
α2-tubulin (Arabidopsis thaliana)	M84696	F: 5′-TCCGCGAAACGAAAATG-3'	[51]
		R: 5'-TGGCTCAAGATCAACAAAGAC-3'	
**RT-qPCR primers used in this study**			
TPS6 Trehalose-6-phosphatase synthase	At1G68020	F: 5'-CTAAAAGCCGAGAAACTCTATCGGACT-3'	[66]
		R: 5'-TCATCGAAAGCATCCTTCTCGCTA-3'	
TPPF (CPuORF26) Trehalose-6-phosphate phosphatase F	At4G12430	F: 5'-TTCTCCACCGCGTAAGAAGC-3'	[67]
		R: 5'-CGCCGAAGGATATTTCACCA-3	
MIOX1 Myo-inositol oxygenase 1	At1G14520	F: 5'-CATGTACCTTGTTGCGAAGGAG-3'	[68]
		R: 5'- ACCATTTTAGCTTGGACGGA -3'	
MIOX2 Myo-inositol oxygenase 2	At2G19800	F: 5'-GCTGTCGTTGGCGATACATTTC-3'	
		R: 5'-AGGGTCGTGCCATTCTTCTTAG-3'	
BAM1 beta-amylase 1	At3g23920	F: 5'-TCAACCTCTTCAACAACCCAC-3'	[69]
		R: 5'-CTCACGGAACAGATCCATCAC-3'	
BAM2 beta-amylase 2	At4g00490	F: 5'-TTTTGGGGCAGAGGACCTGATA-3'	[70]
		R: 5'-GAGCCTCTGGGAAATCCTCATGT-3'	
BAM3 beta-amylase 3	At4g17090	F: 5'-AAAGCACGGTCTCAAACTCCAG-3'	[71]
		R: 5'-ACTGCAAGAGTCTCCTACGTTTCC-3'	
BAM4 beta-amylase 4	At5G55700	F: 5'-GGTGTTCATGGAATCGCAGTTG-3'	[70]
		R: 5'-GCCACCGAACAAAGGAAGTTGA-3'	
AMY3 alpha-amylase-like 3	At1g69830	F: 5'-CGGTGGAGGTAACCACAGAACA-3'	
		R: 5'-AAACTGGCTGCGGAGGCATA-3'	
ISA3 isoamylase 3	At4g09020	F: 5′-GCATCTACAATGACGGAGACGAAA-3′	
		R: 5′-GCCATTCTCCAGGACCATCAAC-3′	
DPE1 D-Enzyme	At5g64860	F: 5'-CCAAAACCCTGCAAATCCTCTG-3'	
		R: 5'-AAAGGCAGTGGCAGAAAGTTCG-3'	
GH3.3 indole-3-acetate-amido synthetase	At2G23170	F: 5'-ACCTATGCTGGGCTTAACCGT-3'	[72]
		R: 5'-AGAGCGATGCGTTCTCAACC-3'	
DRM2 Dormancy/auxin associated family protein 2	At2G33830	F: 5'-GATCGGCGCGTAAGGAAAAC-3'	[73]
		R: 5'-CCATTCCTCTAGTGGCGATGTTA-3'	
β-ACTIN2,7,8	Universal forward	F: 5'-AGTGGTCGTACAACCGGTATTGT-3'	[58]; [57] [56];[55]
β-ACTIN 2	At3G18780	F: 5'-GATGGCATGGAGGAAGAGAGAAAC-3'	
β-ACTIN 7	At5G09810	F: 5'-GAGGAAGAGCATTCCCCTCGTA-3'	
β-ACTIN 8	At1G49240	F: 5'-GAGGATAGCATGTGGAACTGAGAA-3'	
